# Comparison of dermal and eschar fibroblasts in full skin equivalents

**DOI:** 10.1111/wrr.70001

**Published:** 2025-02-12

**Authors:** Gizem Coşar Kutluoğlu, Marcel Vlig, Anouk Elgersma, Bouke K. H. L. Boekema, Willeke F. Daamen, Claudia Doberenz, Dominique Manikowski

**Affiliations:** ^1^ Innovation, Development and Regulatory Affairs MedSkin Solutions Dr. Suwelack Billerbeck Germany; ^2^ Department of Medical BioSciences, Radboud Institute for Medical Innovation Radboud University Medical Center Nijmegen The Netherlands; ^3^ Burn Research Lab Alliance of Dutch Burn Care (ADBC) Beverwijk The Netherlands; ^4^ Plastic, Reconstructive and Hand Surgery Amsterdam UMC Location Free University Amsterdam The Netherlands

**Keywords:** burn, contraction, primary human cells, skin models, wound healing

## Abstract

Full‐thickness burn wounds pose significant problems, demanding specialised therapies to avoid complications and promote recovery. Eschar tissue, which forms in response to severe burns, contains viable fibroblasts, which migrate from the surrounding tissue in response to burn injury and exhibit a myofibroblast phenotype. The goal of this study was to characterise eschar‐derived fibroblasts and examine their use for engineered in vitro full skin equivalents in comparison to normal dermal fibroblasts, which were harvested from non‐injured skin. Microarray analysis indicated that eschar fibroblasts differ from dermal fibroblasts in various biological processes including inflammation, extracellular matrix formation, cell migration and differentiation. Skin equivalents with eschar fibroblasts showed similarities to those generated using normal dermal fibroblasts in terms of epidermis and dermis formation. However, in contrast to dermal fibroblast‐based full skin equivalents, eschar fibroblast‐based equivalents exhibited macroscopic contractile behaviour. In addition, eschar fibroblasts‐based equivalents demonstrated higher *alpha‐smooth muscle actin* expression on mRNA and protein levels. In conclusion, our findings suggest that eschar fibroblasts‐based full skin equivalents hold promise as a platform to study burn wound environments as eschar fibroblasts are clinically more relevant fibroblasts and able to mimic certain aspects of the challenging wound environment in vitro.

## 1 INTRODUCTION

Full‐thickness burn wounds are severe thermal injuries affecting all layers of the skin, including the epidermis, dermis and sometimes underlying tissues. These wounds pose unique challenges and require specialised treatment. Effective management prevents complications such as infection, impaired function and long‐term cosmetic and functional problems. Over time, various treatment options have been developed to address these wounds' complexities and to promote healing.^1^, ^2^ One potential treatment for full‐thickness wounds is the use of acellular dermal templates together with autograft skin. These dermal templates act as temporary scaffolds to facilitate cell attachment and proliferation. MatriDerm®, a collagen‐elastin dermal template, is one example of such a template.^3^ Another option is to combine acellular dermal templates with cells before application to the wound, which may enhance wound healing.^4^, ^5^ Although current treatment options promote wound healing, undesirable conditions such as contraction and scar formation remain a problem. Therefore, it is important to understand the underlying mechanisms to improve full‐thickness burn wound healing.

Fibroblasts play a crucial role in the healing process,^6^, ^7^, ^8^ making it important to closely examine their behaviour in this environment. Dermal fibroblasts are responsible for wound remodelling by synthesising, and degrading extracellular matrix (ECM) components such as collagen and elastin. Myofibroblasts, a subset of fibroblasts involved in wound healing, are contractile cells that are characterised by *alpha‐smooth muscle actin* (*αSMA*) expression. While they help the wound to close or reduce in size and contribute to ECM synthesis, their contractile properties can lead to excessive contraction and fibrosis.^9^, ^10^, ^11^

Eschar tissue is a necrotic tissue that forms after severe burns and is debrided as part of the treatment. Debrided eschar tissue contains viable fibroblasts that exhibit similar characteristics to myofibroblasts such as *αSMA* expression and contractility. Although the origin of these eschar‐derived fibroblasts is unknown, they are believed to be a population of stem cells that migrate from the surrounding tissue of the wound bed in response to burn injury. After debridement, these fibroblasts may remain or regenerate in the wound bed and cause contraction with their myofibroblast‐like phenotype.^12^, ^13^ Additionally, it has been shown that upon injury, cells are capable of sensing mechanical changes in their microenvironment that may lead to the differentiation of fibroblasts into myofibroblasts.^14^ These *αSMA*‐expressing cells maintain their myofibroblast‐like phenotype over an extended period of time, as they ‘remember’ the mechanical cues from the wound environment.^15^ This makes eschar fibroblasts a clinically relevant source to study wound conditions in vitro, as their phenotype can be maintained over time by recalling the specific characteristics of the wound microenvironment. Additionally, these cells can be easily obtained from discarded wound material.

Full skin equivalents (FSEs) are bioengineered skin constructs that replicate the structure and function of human skin, including the epidermis, dermis and, in some instances, the hypodermis. FSEs mimic the structure and the function of natural skin by combining different cell types embedded in a biomaterial scaffold, for example, ECM components.^16^, ^17^ These sophisticated tissue constructs offer a wide range of uses, including skin biology research, product testing, animal testing alternatives and treatment of patients.^16^, ^18^, ^19^ Primary human dermal fibroblasts co‐cultured with primary human keratinocytes have been used to generate FSEs on MatriDerm®,^20^ which mimic the structure of ex vivo human skin in terms of the epidermal and dermal layers. Furthermore, 2 weeks after burn induction in vitro, re‐epithelisation and high levels of pro‐inflammatory cytokines were measured in these FSEs, similar to ex vivo human skin. This indicates that MatriDerm®‐based FSEs may be used as a platform to study burn wound healing in a more relevant setting.^20^

Given their myofibroblast‐like phenotype and potential to reproduce the wound environment, the application of eschar fibroblasts holds considerable importance. We hypothesised that eschar fibroblasts, as wound‐derived fibroblasts, would have different gene expressions than unwounded dermal fibroblasts and could be used as a model to study wound microenvironment in vitro. To test our hypothesis, gene expression profiles of dermal and eschar fibroblasts from the same donor were compared using microarray analysis. Subsequently, FSEs were generated on MatriDerm® using eschar fibroblasts and compared with those produced with primary dermal fibroblasts.

## 2 MATERIALS AND METHODS

### 2.1 Cell isolation and propagation

For the microarray analysis, dermal fibroblasts were isolated from surplus autologous skin grafts, while eschar fibroblasts were isolated from debrided tissue from the same donor (*n* = 10) treated for burns at the burn centre of the Red Cross Hospital in Beverwijk, The Netherlands. For FSEs, dermal fibroblasts and keratinocytes were isolated from healthy skin samples collected during abdominal reconstructive surgery at the Plastic and Reconstructive Surgery Department of the same hospital. Eschar fibroblasts were isolated from debrided tissues of patients treated for burns at the burn centre of the same hospital. Consent for the use of these anonymised, post‐operative residual tissue samples was received through the informed opt‐out protocol of the Red Cross Hospital, which was in accordance with the national guidelines (https://www.coreon.org/, accessed on 23 November 2020) and approved by the institutional privacy officers. Subjects were actively informed of this procedure and were able to withdraw at any point easily. See Table 1 for relevant donor information.

**TABLE 1:** Donor information included skin samples, where healthy donor information corresponds to dermal fibroblasts and keratinocytes. The total number of donors was 23; 10 donors for microarray analysis and 13 donors for the preparation of FSE models. To ensure the anonymity of patient data, age, and total body surface area (%TBSA), post‐burn day (PBD) was categorised.

	Donor	Age (years)	Sex	%TBSA	PBD	Origin	Passage Nr.
Fibroblasts for Microarray analysis	1	18–65	F	21–40	15–21	Dermal and eschar	2
	2	0–17	M	<20	15–21	Dermal and eschar	2
	3	65+	M	<20	>21	Dermal and eschar	2
	4	18–65	F	<20	>21	Dermal and eschar	2
	5	18–65	M	>40	>21	Dermal and eschar	2
	6	0–17	M	<20	0–14	Dermal and eschar	2
	7	18–65	F	<20	15–21	Dermal and eschar	2
	8	18–65	M	<20	0–14	Dermal and eschar	2
	9	18–65	M	<20	0–14	Dermal and eschar	2
	10	18–65	F	<20	15–21	Dermal and eschar	2
Fibroblasts for FSE	11	U	U	n.a.	n.a.	Dermal (FSE model 1)	2
	12	18–65	F	n.a.	n.a.	Dermal (FSE model 2)	2
	13	65+	M	n.a.	n.a.	Dermal (FSE model 3)	2
	14	18–65	F	n.a.	n.a.	Dermal (FSE model 4)	2
	15	65+	M	U	0–14	Eschar (FSE model 1)	2
	16	0–17	M	U	0–14	Eschar (FSE model 2)	3
	17	18–65	M	U	0–14	Eschar (FSE model 3)	2
	18	U	U	U	0–14	Eschar (FSE model 4)	2
Keratinocytes for FSE	19	U	U	n.a.	n.a.	Dermal (Used for Dermal FSE model 1)	3
	20	18–65	F	n.a.	n.a.	Dermal (Used for Eschar FSE model 1)	3
	21	18–65	F	n.a.	n.a.	Dermal (Used for Dermal & Eschar FSE model 2)	1
	22	18–65	F	n.a.	n.a.	Dermal (Used for Dermal & Eschar FSE model 3)	1
	23	18–65	M	n.a.	n.a.	Dermal (Used for Dermal & Eschar FSE model 4)	1

Abbreviations: F, female; M, male; n.a, not applicable; U, unknown.

A dermatome (Aesculap AG & Co. KG; Tuttlingen, Germany) was used to collect 0.3 mm thick split‐thickness samples from healthy skin. The epidermis and dermis were separated by incubating the skin in 0.25% (w/v) dispase II (1.8 units/mg; Gibco; Waltham, USA) for 45 min at 37°C. Dermal fibroblasts were isolated by cutting the dermis into small pieces and immersing these in a solution containing 0.25% (w/v) collagenase A (0.24 units/mg; Roche; Penzberg, Germany) and 0.25% (w/v) dispase II for 2 h at 37°C. The cell suspension was supplemented with 1 mM EDTA in phosphate‐buffered saline (PBS) to inhibit collagenase, passed through a 500 μm cell strainer and centrifuged at 360 × g for 10 min. The cell pellet was resuspended in fibroblast medium (FBM; Dulbecco's modified Eagle's medium (DMEM; Gibco; Waltham, USA) supplemented with 10% FetalClone III (HyClone; Logan, USA), 1% Glutamax (Gibco; Waltham, USA), and 1% antibiotics (100 IU/mL penicillin, 100 mg/mL streptomycin; Invitrogen; Waltham, USA). The suspension was filtered through a 70 μm cell strainer and cultured in flasks at 37°C in 5% CO_2_. All cell cultures were performed at 37°C in 5% CO_2_. Eschar fibroblasts were isolated from debrided tissue using the same fibroblast isolation procedure.

To isolate keratinocytes, the epidermis was incubated in 0.05% (w/v) trypsin–EDTA (Gibco; Waltham, USA) for 20 min at 37°C. The obtained cell suspension was passed through a 70 μm cell strainer and centrifuged at 110 × g for 10 min. The cell pellet was resuspended in FBM and centrifuged at 160 × g for 10 min. Subsequently, the cell pellet was resuspended in CnT‐07 (CellnTec; Bern, Switzerland) culture medium and cultured in 1 μg/cm^2^ type IV collagen (Sigma‐Aldrich; St. Louis, USA) coated flasks. The medium was changed twice a week.

### 2.2 Microarray analysis

Dermal and eschar fibroblasts, isolated from autologous skin and eschar tissue of the same donor, respectively, were cultured separately until passage 2. After 2 days of culture in a culture flask, total RNA was isolated using the TRIzol™ Reagent (Invitrogen; Waltham, USA) according to the manufacturer's protocol. RNA concentration was determined using the Nanodrop Spectrophotometer (Thermo Scientific; Waltham, USA). Microarray analysis was conducted following the protocol described in.^21^ Briefly, 1 μg cRNA was labelled using the Agilent protocol (Agilent Technologies; Santa Clara, USA). cRNA concentration and labelling efficiency were determined using a NanoDrop ND‐1000 UV–vis Spectrophotometer (NanoDrop Technologies; Wilmington, USA). From each donor, labelled cRNA was hybridised for 15–17 h at 65°C on 44 K Human Agilent whole genome arrays according to the manufacturer's protocol. After washing, arrays were quick‐dried using acetonitrile. Arrays were scanned using the Agilent G2505B GeneArray Scanner (Agilent Technologies; Santa Clara, USA) with default settings for two‐colour hybridisation. Agilent Feature Extraction (v8.0) software was used to extract signal intensities. Normalisation of gene expression data was performed within the R statistical software using the linear models for microarray data (Limma), consisting of background subtraction, within‐array normalisation (Loess) and between‐array normalisation (Aquantile). Moderated t‐tests using empirical Bayes variance estimation implemented in Limma were used to assess the difference in gene expression between the dermal and eschar fibroblasts. The Benjamini–Hochberg procedure was applied to the raw p‐values to control for false discovery rate (FDR) to address the multiplicity problem due to the large number of genes tested. Data from the microarray was sorted to identify genes with a log Fold Change (logFC) of > − 2 or >2 with a significant p‐value of ≤0.05. This set of genes was imported into DAVID 6.7, an online tool for functional annotation. DAVID was used to look for enriched KEGG pathways.^22^, ^23^

Fifteen pathways potentially involved in the biology of the wound were selected from the list of pathways defined in the KEGG repository.^24^, ^25^ The probes on the array were mapped to the genes comprising the pathway. The Globaltest was performed to assess whether the global expression pattern is significant between groups using the gene expression data from these probes.^26^

### 2.3 Full skin equivalents

**FIGURE 1:**
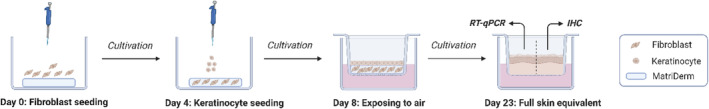
Full Skin Equivalent Generation. On day 0, fibroblasts were seeded on scaffolds and 4 days later, keratinocytes were seeded on top, and culture was continued. On day 8, cultures were exposed to air and cultured until harvesting on day 23. At the end of the culture, one‐half of FSE was used for real‐time quantitative PCR (RT‐qPCR), and the other half was used for immunohistochemistry (IHC). Created in BioRender. Daamen, W. (2024) BioRender.com/v88w425.

Keratinocytes, dermal fibroblasts and eschar fibroblasts from different donors (Table 1) were used to generate the FSEs. FSE model generation was based on a published protocol^20^ and is visualised in Figure 1. In detail, sterile MatriDerm® (2 mm thickness; MedSkin Solutions Dr. Suwelack AG, Billerbeck, Germany) was punched into 12 mm diameter circular pieces. On day 0, 2 × 10^5^ fibroblasts were seeded onto the MatriDerm® and the matrices were submerged in an FBM culture medium containing 0.2% ascorbic acid (Sigma‐Aldrich; St. Louis, USA) for 4 days. Next, 1 × 10^5^ keratinocytes were seeded on top of the fibroblasts, and the models were cultured submerged in DMEM/Ham's F12 Nutmix (3:1) (Invitrogen; Paisley, UK) containing 5% FetalClone III, 1% antibiotics (100 IU/mL penicillin, 100 mg/mL streptomycin), 1.1 μM hydrocortisone (Sigma‐Aldrich; St. Louis, USA), 1 μM isoproterenol (Sigma‐Aldrich; St. Louis, USA), 0.09 mM bovine insulin (Sigma‐Aldrich; St. Louis, USA), 2 ng/mL keratinocyte growth factor (KGF; ImmunoTools; Friesoythe, Germany) and 0.5 ng/mL epidermal growth factor (EGF; ImmunoTools; Friesoythe, Germany). On day 8, the FSEs were transferred to transwell cell culture inserts and cultured air exposed in DMEM/Ham's F12 Nutmix (3:1) containing, 2% FetalClone III, 1% antibiotics (100 IU/mL penicillin and 100 mg/mL streptomycin), 1.1 μM hydrocortisone, 1 μM isoproterenol, 0.09 μM bovine insulin, 0.2% lipid supplement (25 mM palmitic acid, 15 mM linoleic acid, 7 mM arachidonic acid and 24 mM bovine serum albumin; all Sigma‐Aldrich; St. Louis, USA), 0.2% Vitamin E in ß‐cyclodextrin (2 μL vitamin E/500 μL ß‐cyclodextrin) (both Sigma‐Aldrich; St. Louis, USA), 4 ng/mL KGF and 1 ng/mL EGF. From day 11, FSEs were cultured in DMEM/Ham's F12 Nutmix (3:1) containing 0.5% FetalClone III, 1% antibiotics (100 IU/mL penicillin and 100 mg/mL streptomycin), 1.1 μM hydrocortisone, 1 μM isoproterenol, 0.09 μM bovine insulin, 10.1 μM carnitine, 9.99 μM serine, 0.2% lipid supplement (25 mM palmitic acid, 15 mM linoleic acid, 7 mM arachidonic acid and 24 mM bovine serum albumin; all Sigma‐Aldrich; St. Louis, USA), 0.4% vitamin E in ß‐cyclodextrin (2 μL vitamin E/500 μL ß‐cyclodextrin), 4 ng/mL KGF, 1 ng/mL EGF, 0.2% ascorbic acid, and the medium refreshed twice a week until day 23. A total of 4 FSEs were generated per fibroblast type and each FSE was divided in half, where one half was used for gene expression analysis and the other half for immunohistochemical analysis.

### 2.4 Real‐time quantitative PCR

To confirm the microarray analysis, cDNA synthesis was performed using the QuantiTect reverse transcription kit (Qiagen; Hilden, Germany). qPCR was performed using Sso Advanced Universal SYBR Green Supermix on a CFX‐connect PCR system (Bio‐Rad; Hercules, USA). The primers are provided in the Supplementary Material (Table S1). Two technical replicates were run for each gene. When the standard deviation of duplicates was higher than 0.3, they were excluded from the calculation to exclude false duplicates. Used results are depicted in graphs showing individual data points for each donor. Normalised expression of qPCR data^27^ was calculated using CFX manager (Bio‐Rad; Hercules, USA).

To analyse the gene expression in the FSEs, the total RNA isolation was conducted using the ReliaPrep™ RNA Miniprep system (Promega; Fitchburg, USA) according to the manufacturer's protocol. The quality and quantity of total RNA in each sample were determined using Spectradrop™ (Molecular Devices; San Jose, USA). cDNA was synthesised using oligo(dT)_15_ and random primers (1:1) supplied with GoScript™ Reverse Transcriptase (Promega; Fitchburg, USA). Later, GoTaq® qPCR Master Mix (Promega; Fitchburg, USA) was used on a CFX Connect Real‐Time System (Bio‐Rad; Hercules, USA). Primers were designed according to^28^ and provided in the Supplementary Material (Table S2). Two technical replicates were run for each gene and when the standard deviation of duplicates was higher than 0.5, they were excluded from the calculation and used data were visualised per donor. Normalised expression was calculated using GraphPad Prism version 10.0.2 (GraphPad Prism Software; San Diego, USA). The sample size was *N* = 4 for dermal‐FSEs and N = 4 for eschar‐FSEs.

### 2.5 Immunohistochemistry

The samples were fixed in kryofix (50% ethanol, 3% PEG300) at 4°C for 2 days and then embedded in paraffin. Subsequently, the samples were cut into 5 μm thick sections and used for haematoxylin & eosin (H&E) or IHC. First, slides were deparaffinised and rehydrated by serial washing in xylene two times for 5 min, 100%, 96% and 70% ethanol for 2 min and de‐ionised H_2_O for 5 min. For H&E staining, slides were placed in Epredia™ Modified Mayers haematoxylin solution (Fisher Scientific; Landsmeer, The Netherlands) for 2 min, rinsed with tap water for 30 min, and placed in Eosin solution for 30 s. After dehydration by serial washing in ascending series of ethanol, xylene I and xylene II for 2 min, sections were mounted with Eukitt Quick hardening medium (Sigma‐Aldrich; St. Louis, USA). The antibodies for IHC are listed in Table 2. For antibody staining, slides were treated with 1% hydrogen peroxide for 15 min at room temperature (RT) for blocking of endogenous peroxidase. Antigen retrieval was performed by incubation in the chemicals shown in Table 2 and then sections were pre‐incubated with 5% normal goat serum (Abcam; Cambridge, UK) diluted in PBS + 1% bovine serum albumin. Sections were then incubated with primary antibodies to detect pan‐cytokeratin, cytokeratin‐10, type IV collagen, type III collagen and αSMA. For dermatan sulphate staining, after endogenous peroxidase blocking, sections were first incubated with GD3A12 antibody^29^ for 1 h, then washed with PBS and incubated with the secondary antibody anti‐VSV P5D4 (1:10 dilution) for 1 h at RT. Sections were then incubated in a polymer‐horse radish peroxidase‐goat‐anti‐mouse antibody or polymer‐horse radish peroxidase‐goat‐anti‐rabbit antibody (Bright Vision; Groningen, The Netherlands) for 30 min at RT. After PBS washing, sections were stained with 3,3′‐diaminobenzidine (DAB) to detect specific antigen–antibody binding. Development of DAB staining was observed under a microscope and optimum staining time was determined and used for all sections. The reaction was stopped by placing sections in de‐ionised H_2_O. The sections were subsequently counterstained with haematoxylin, dehydrated and mounted with Eukitt Quick hardening medium.

**TABLE 2:** Primary antibody used in this study.

Primary antibody	Clone	Cat. Nr.	Host	Dilution	Manufacturer	Antigen retrieval
Pan‐cytokeratin	C‐11 + PCK‐26 + CY‐90 + KS‐1A3 + M20 + A53‐B/A2	C2562	Mouse	1:500	Sigma	0.1% Triton‐X‐100, 5 min
Cytokeratin 10	Polyclonal	ab111447	Rabbit	1:500	Abcam	0.1% Triton‐X‐100, 5 min
Type IV collagen	CIV 22	M0785	Mouse	1:100	DAKO	0.1% Triton‐X‐100, 5 min
Dermatan Sulphate	GD3A12	n.a.		1:20	29	n.a.
Type III collagen	FH‐7A	ab6310	Mouse	1:200	Abcam	10 mM Sodium citrate pH 610 min 70°C
αSMA	1A4	M0851	Mouse	1:500	DAKO	10 mM Tris‐EDTA pH 9, 10 min 70°C

### 2.6 Microscopy

Microscopic imaging was performed using a Panoramic 250 Flash III (3DHISTECH, Budapest, Hungary) fully automated digital microscope. Images were reviewed using CaseViewer version 2.4 (3DHISTECH; Budapest, Hungary). Thickness measurements of epidermal and dermal layers in H&E‐stained samples were performed using Fiji software. Five points were randomly selected to measure the thickness of the epidermis and dermis separately. For each FSE, five different measurements were averaged and presented in the graph.

### 2.7 Statistical analysis and data visualisation

Statistical analysis was performed using GraphPad Prism version 10.0.2 (GraphPad Prism Software; San Diego, USA). The Mann–Whitney U test was used to determine differences. Differences were noted as follows: ns = not significant; *p ≤ 0.05; **p ≤ 0.01; ***p ≤ 0.001.

## 3 RESULTS

### 3.1 Dermal and eschar fibroblasts show distinct gene expression profiles

**FIGURE 2:**
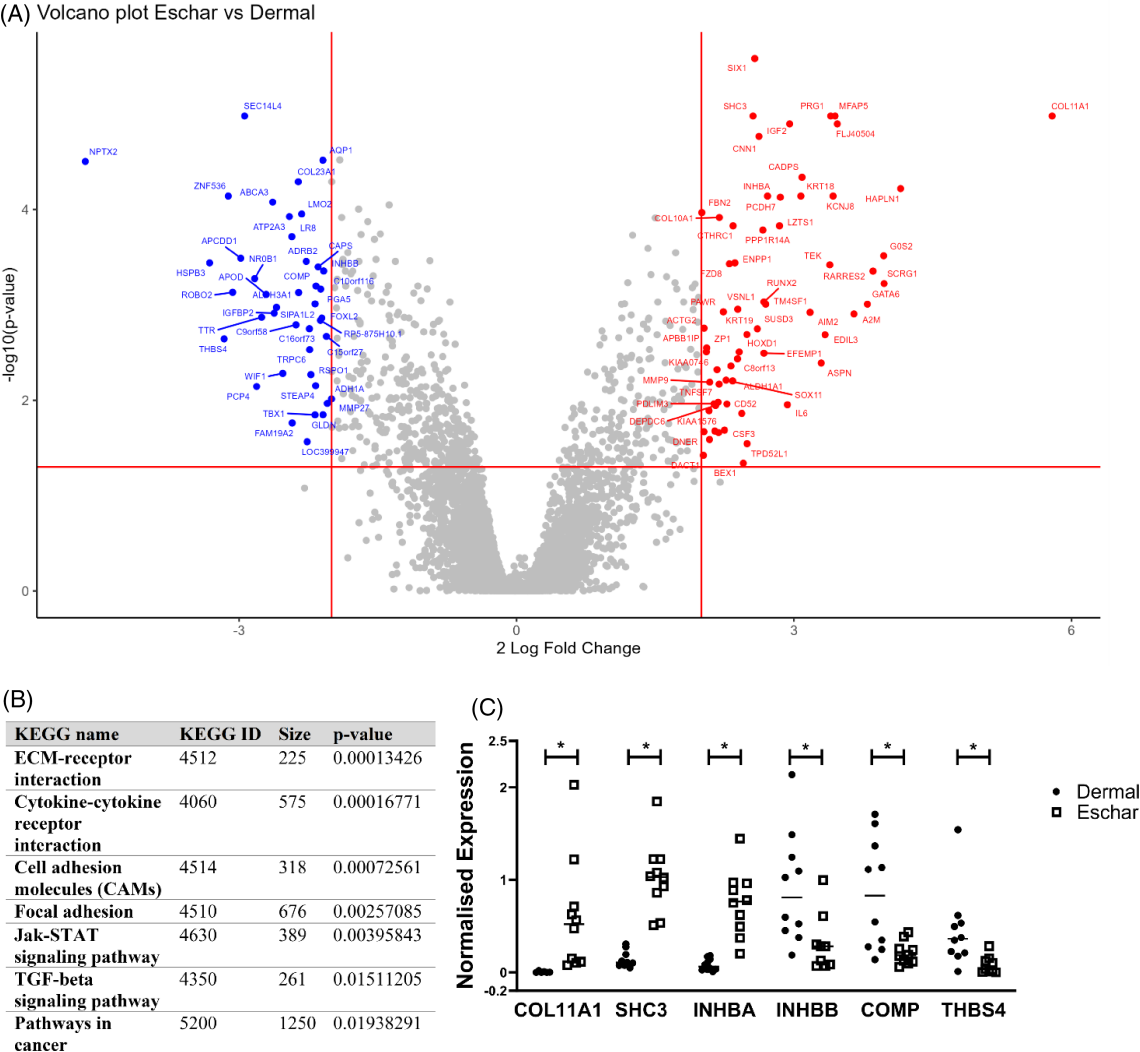
Microarray analysis of dermal and eschar fibroblasts. A) Volcano plot of differentially expressed genes in eschar vs. dermal fibroblasts identified by microarray analysis (*n* = 10). All 93 genes that showed a significant difference in expression with a fold change greater than 2 (Log2(fold change) < −2 or >2) are shown. Only the genes with a fold change >2 have been labelled. Gene names in red are upregulated in eschar fibroblasts, whereas gene names in blue are downregulated. B) Differentially expressed pathways between eschar and dermal fibroblasts. C) RT‐qPCR analysis to verify microarray results by testing 6 genes that were significantly different between eschar (*n* = 10) and dermal fibroblasts (n = 10). Each point represents an individual donor, and the mean is shown as a horizontal line. *COL11A1; n* = 6 for dermal, n = 10 for eschar, *SHC3*; *n* = 9 for dermal, n = 10 for eschar, *INHBA*; n = 10 for dermal and eschar, *INHBB*; n = 10 for dermal, n = 9 for eschar, *COMP*; n = 10 for dermal and eschar, *THBS4*; n = 10 for dermal, n = 9 for eschar. Statistical analysis was performed with Mann–Whitney U test; Differences were noted as *p ≤ 0.05.

First, we analysed differential gene expressions in dermal and eschar fibroblasts cultured in cell culture dishes. Microarray analysis revealed that 93 genes out of the 17,434 genes examined showed significant differences in expression between eschar and dermal fibroblasts from the same donor (Figure 2A) (*p* ≤ 0.05). The complete list of genes and raw data can be accessed from Mendeley database (DOI:10.17632/7by2533r67.1). Pathway analysis using the KEGG pathway database^24^ was utilised to identify pathways altered in dermal and eschar fibroblasts by differentially expressed genes (Figure 2B). Among these, ECM receptor interaction, focal adhesion and TGF‐beta signalling pathways are particularly relevant to this study aiming to mimic human wounded skin in vitro and investigate tissue remodelling mechanism. To verify microarray data, some of the significantly altered genes in pathways of interest were tested by RT‐qPCR (Figure 2C). Results confirmed that *COL11A1*, a fibrillar collagen that regulates fibrillogenesis, *SHC adaptor protein 3 (SHC3)* and *inhibin A (INHBA)*, which induces cell proliferation and type I collagen production,^30^ were upregulated in eschar fibroblasts. On the other hand, *cartilage oligomeric matrix protein (COMP)*, *inhibin B (INHBB)*, and *thrombospondin 4 (THBS4)*, which are all markers of ECM degradation and ECM organisation were downregulated. This indicates that eschar fibroblasts contribute to ECM production rather than ECM degradation compared to skin fibroblasts.

### 3.2 Dermal and eschar fibroblasts‐based FSEs resemble human skin with structured epidermis and dermis

**FIGURE 3:**
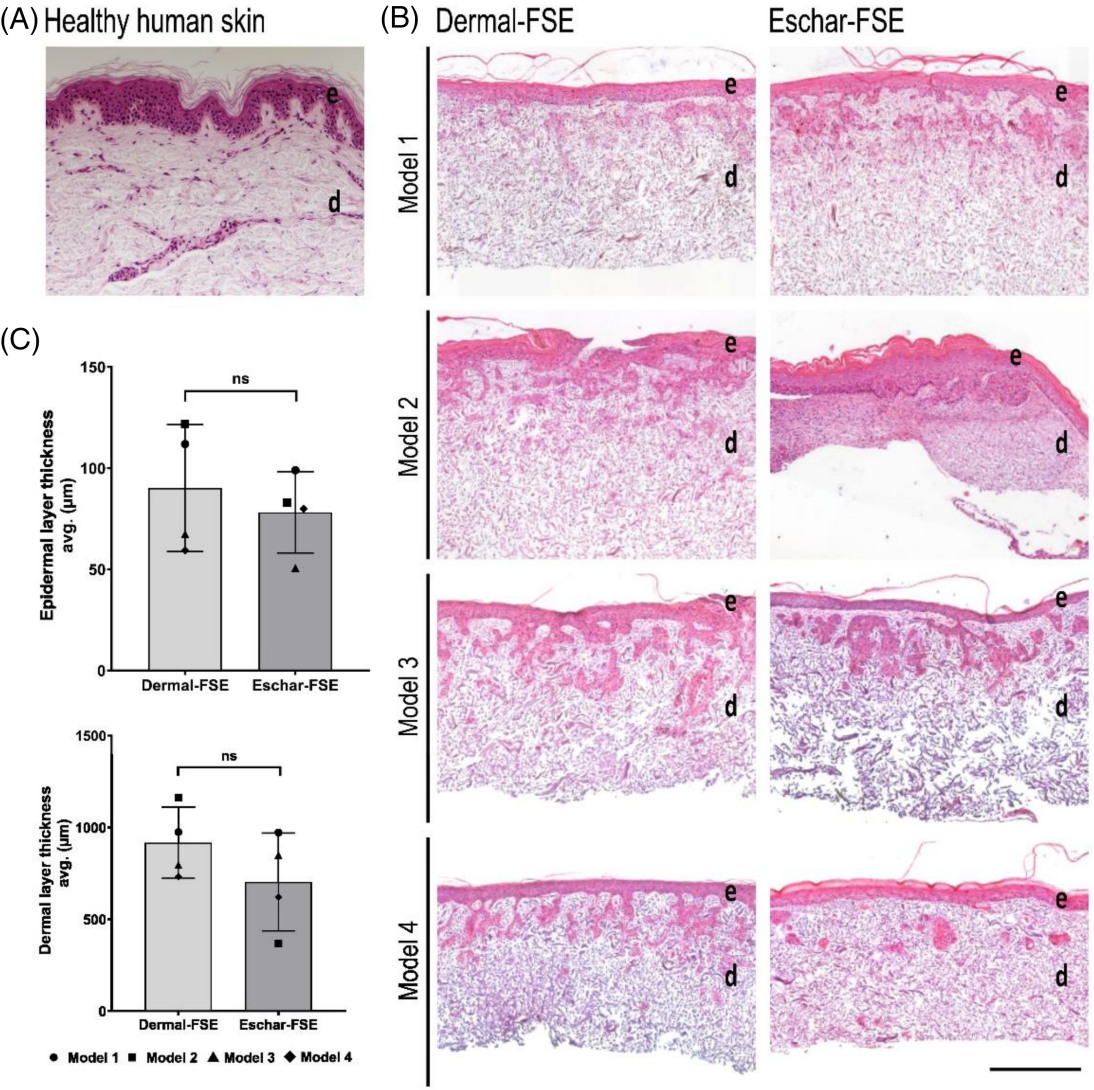
FSEs were successfully generated with dermal and eschar fibroblasts. A) H&E staining of human skin. B) H&E stained dermal‐FSE and eschar‐FSE of each model show proper epidermal (e) and dermal (d) layers formation on both dermal‐FSEs and eschar‐FSEs. C) Bar graphs present the measured thickness of the epidermis and dermis in FSEs. *n* = 4 for dermal‐ and eschar‐FSEs. Statistical significance was determined with the Mann–Whitney U test; differences were noted as follows: Ns = not significant. Scale bar: 500 μm.

To further investigate the differences and similarities of dermal and eschar fibroblasts, we used a three‐dimensional (3D) engineered skin equivalent model, which has been previously demonstrated to mimic full thickness skin in vitro.^20^ Despite the distinct gene expression profiles found in microarray analysis, both fibroblast types successfully generated 3D FSEs. The epidermis and dermis formed as two distinct layers, with the epidermis completely covering the dermis, similar to human skin (Figure 3A,B). The thickness of the epidermis was similar between FSEs, 90 ± 31 μm for dermal‐FSEs and 78 ± 20 μm for eschar‐FSEs, and was within the published range for epidermal thickness of normal human skin, i.e. 77 ± 26–267 ± 120 μm.^31^ Dermal layer thicknesses decreased during culture from 2 mm to 0.9 ± 0.2 mm and 0.7 ± 0.3 mm for dermal fibroblast‐based FSEs (dermal‐FSEs) and eschar fibroblast‐based FSEs (eschar‐FSEs), respectively (Figure 3C).

### 3.3 mRNA expression influenced by culture dimensionality and donor variability

**FIGURE 4:**
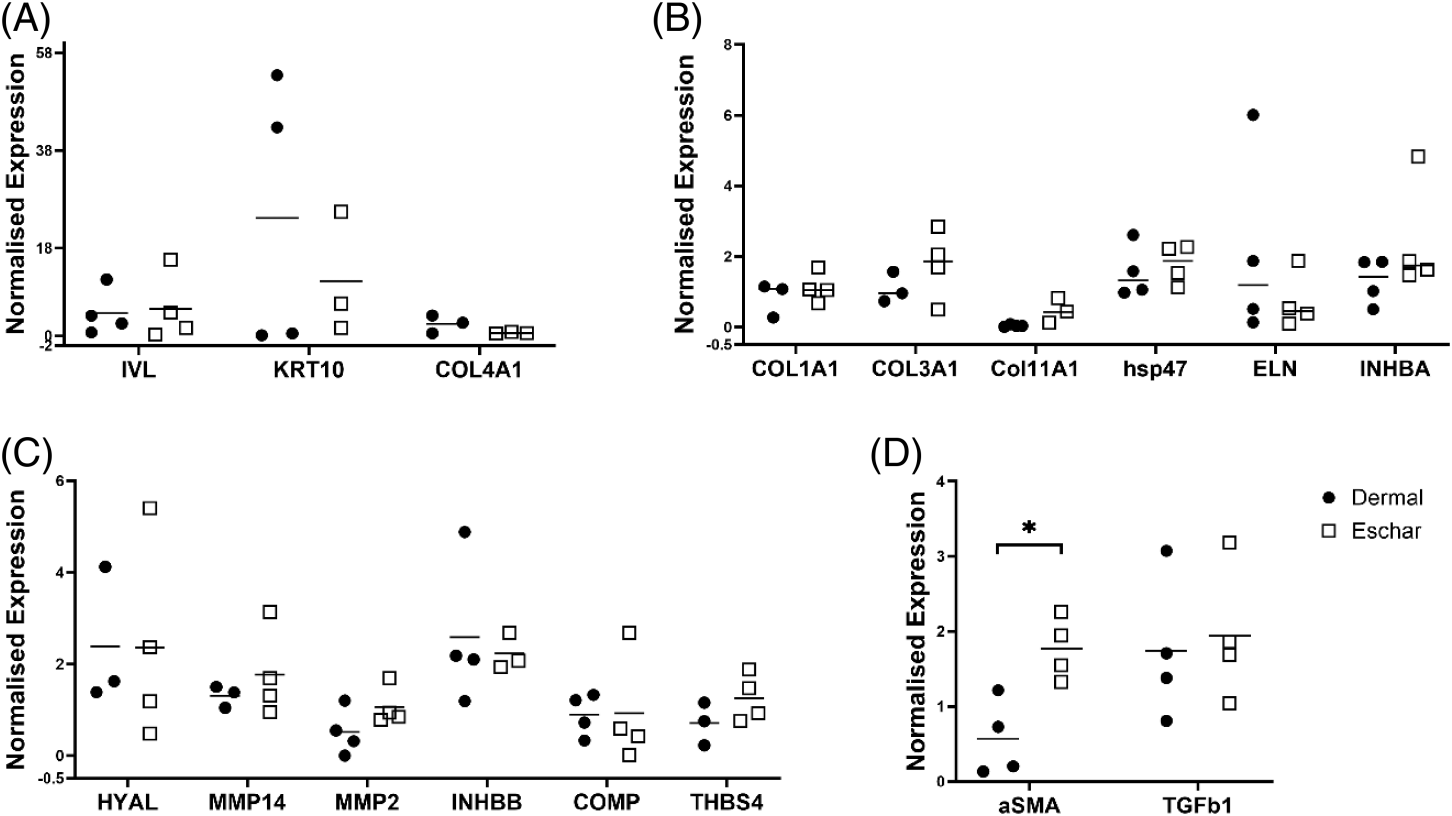
Gene expression levels in eschar‐FSEs differ significantly from dermal‐FSEs with higher *αSMA* expression. (A) genes for the epidermis and basement membrane formation, (B) genes for ECM deposition, *COL11A1*; *n* = 4 for dermal, (C) genes for ECM degradation and (D) genes for contraction. Full gene names can be found in Table S2. Normalised gene expressions were represented as mean and standard deviation. The Mann–Whitney *U* test was used to determine differences in RT‐qPCR data. Differences were noted as *p ≤ 0.05.

Next, we investigated whether the differentially expressed genes found in the microarray are also differentially expressed in 3D FSEs. None of the previously affected genes were differentially expressed in 3D (Figure 4B,C). We then studied other epidermis formation, ECM deposition and degradation, and myofibroblast differentiation markers on the mRNA level. For epidermis formation, the genes *involucrin (IVL)* and *keratin 10 (KRT10)*, which are early differentiation markers, and *COL4A1*, a basement membrane marker, were tested. As expected, no differences between dermal‐ and eschar‐FSEs were observed for these markers since keratinocytes from non‐injured skin biopsies were used for all FSEs (Figure 4A).

Type I and III collagen are the major constituents of the skin, and their replacement is paramount. Therefore, we analysed the synthesis of *COL1A1* and *COL3AI* and found similar levels between dermal‐ and eschar‐FSEs. *SERPINH1 encoding heat shock protein 47 (hsp47)*, a collagen chaperone playing a role in collagen maturation, was also expressed similarly between FSEs. Elastin, another important ECM protein, provides elasticity to tissue but is difficult to rebuild when injured.^32^ *Elastin (ELN)* synthesis showed a trend of downregulation in eschar‐FSEs, however, it was not differentially expressed in eschar‐FSEs (Figure 4B).

Injuries cause changes in ECM composition and structure, and stimulate the production of ECM degrading enzymes to remodel the tissue.^33^ Since eschar fibroblasts are isolated from the wound environment, ECM degradation genes are expected to show an altered expression in comparison to dermal fibroblasts. To test this hypothesis, hyaluronidase 1 (HYAL), *matrix metalloproteinase‐2 (MMP2)*, and *matrix metalloproteinase‐14 (MMP14)* were analysed. Among these genes, *MMP2* showed a trend of upregulation, however, results were not significantly different (Figure 4C). In summary, we did not detect any differential gene expression patterns for ECM deposition or degradation when eschar fibroblasts are cultured in FSE compared to dermal fibroblasts.

Since eschar fibroblasts were described to be more prone to differentiate into myofibroblast‐like phenotypes,^12^ we also investigated *αSMA* expression levels. Although no significant differences were found using microarray analysis for two‐dimensional cell cultures, *αSMA* expression was considerably increased in eschar‐FSEs (Figure 4D). Furthermore, despite the significant changes in the TGF‐beta signalling pathway in eschar fibroblasts (Figure 2B), *TGFβ1* was not increased in eschar‐FSEs.

### 3.4 Eschar fibroblasts contract the dermal template in FSEs

**FIGURE 5:**
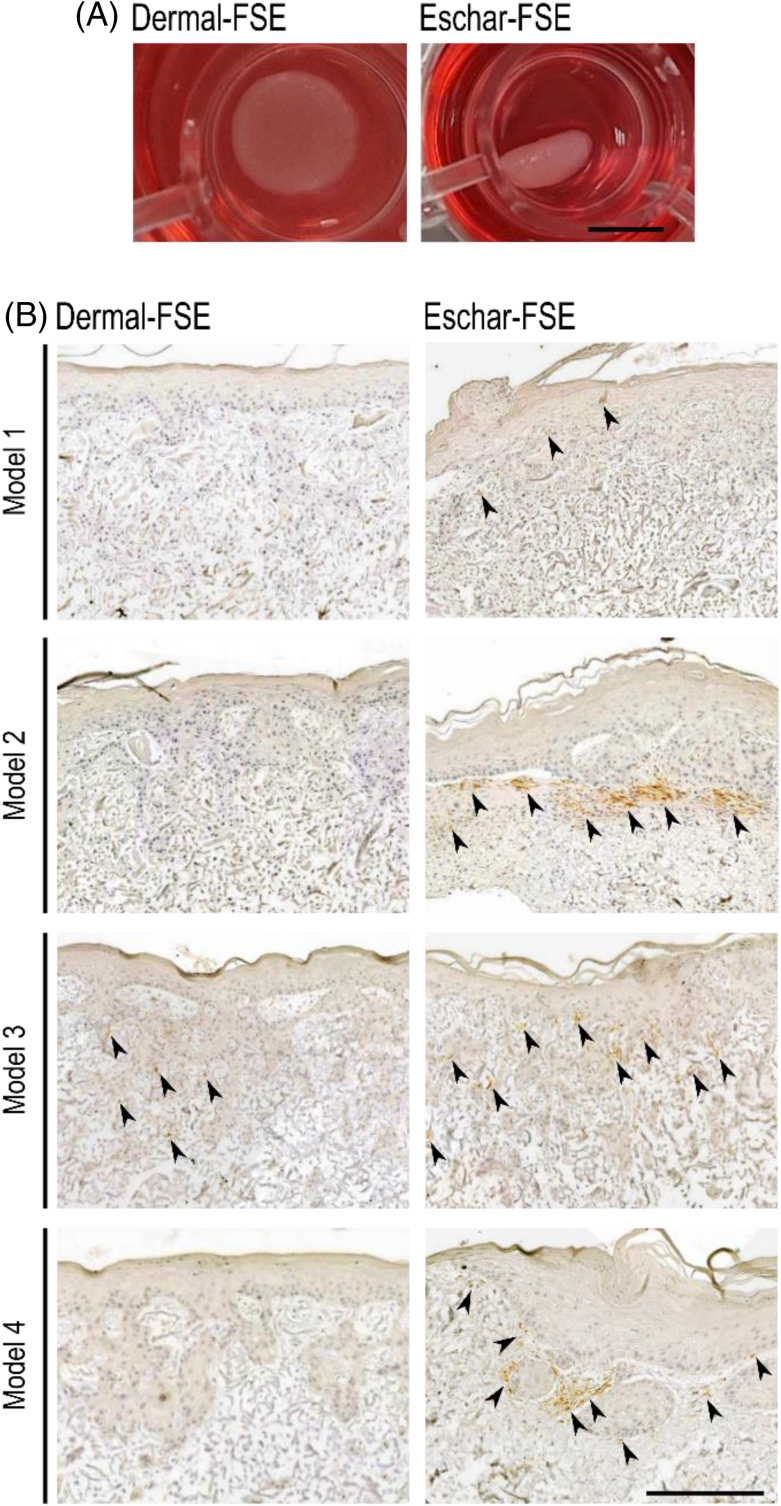
Eschar‐FSEs showed contractile behaviour, as evidenced by (A) their ability to curl the matrix (it was similar for all the donors with varying degrees, picture from Model 1) and (B) positive αSMA staining highlighted with arrowheads. *n* = 4 for each fibroblast type. Scale bars: 6 mm in A, 200 μm in B.

Next, we investigated dermal‐ and eschar‐FSE on protein levels. It was visible at a first glance that in comparison to dermal‐FSE, eschar‐FSE curled up during culture (Figure 5A). Eschar‐FSE matrix curling in vitro might mimic wound contraction in healing skin in vivo. Wound contraction is known to impair healing, can lead to scarring and is often associated with *αSMA* expression by myofibroblasts.^34^ Curling of eschar‐FSEs may be due to eschar fibroblasts expressing higher levels of αSMA than dermal fibroblasts. Indeed, in line with macroscopic observations and RT‐qPCR, all eschar‐FSEs showed positive αSMA protein staining, mostly in a layer below the epidermis with varying degrees (Figure 5B). Dermal‐FSEs were mainly negative for αSMA, except for dermal‐FSE Model 3, which showed some positive staining.

### 3.5 Epidermal and dermal layers formed similarly in both dermal‐ and eschar‐FSEs

**FIGURE 6:**
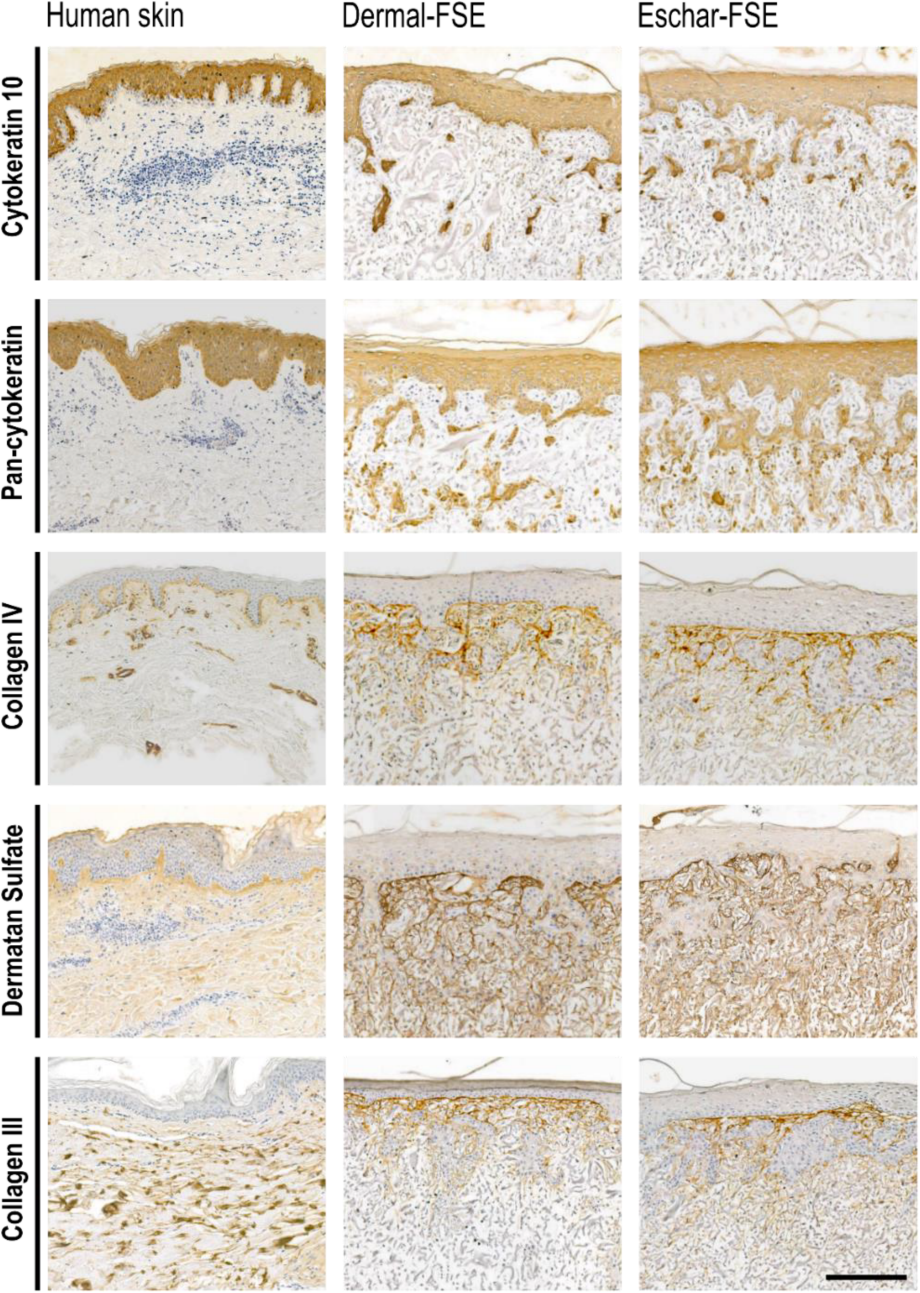
Epidermal and dermal layers were similarly formed in all FSEs after 23 days of culture. Results are from Model 1 and show staining for cytokeratin 10, pan‐cytokeratin, type IV collagen, type III collagen and dermatan sulphate. The same orientation was used for the images with the epidermis facing upwards. In the absence of primary antibodies, staining remained negative in all samples. *n* = 4 for each fibroblast type. Scale bar: 200 μm.

In skin wound healing, epidermis and dermis formation are required for wound closure and tissue replacement, respectively. To assess the epidermis and dermis development after 23 days of culture, immunohistochemical stainings were performed, as shown in Figure 6. The epidermal layer formation was assessed by staining for cytokeratin 10, a marker of early keratinocyte differentiation, and pan‐cytokeratin, a cocktail of various cytokeratin antibodies. In line with RT‐qPCR data, no differences were found in the formation of an epidermal layer in both dermal‐ and eschar‐FSEs. As expected, the basement membrane marker type IV collagen was synthesised mainly underneath the epidermal layer with limited variation between FSEs. However, keratinocytes cultured on a porous matrix not only grow on top of the matrix but also clustered in the dermal layer, resulting in disorganised basement membrane between the epidermal and dermal layers, in contrast to the fine line present in human skin.

The dermal layer mainly consists of type I collagen.^35^ Since it cannot be distinguished between MatriDerm®‐derived collagen and newly synthesised collagen via direct antibody‐staining, dermatan sulphate was used as a marker for newly synthesised collagen. Dermatan sulphate is a collagen‐fibril‐associated glycosaminoglycan, and MatriDerm® and other purified collagens do not contain any residual dermatan sulphate^29^ (Supplementary Figure S2). Therefore, we used dermatan sulphate as a proxy for type I collagen synthesis. As expected, dermatan sulphate staining showed uniform collagen synthesis in the dermis of all FSEs, and in line with RT‐qPCR data, no differences between dermal and eschar‐FSE were detected. Type III collagen is the second most abundant collagen in skin, synthesised during early wound healing and is subsequently substituted by type I collagen.^36^ We therefore assessed the levels and expression pattern of type III collagen in FSEs. Both FSEs exhibited comparable expressions of type III collagen, which verifies the RT‐qPCR data. In comparison to human skin, type III collagen expression was mainly present in the papillary layer, beneath the epidermis in FSEs. Overall, we found that eschar fibroblasts exhibit an ECM‐producing phenotype compared to dermal fibroblasts in 2D, which was less pronounced in 3D FSEs. However, the myofibroblast‐like phenotype was of greater significance in 3D, which contracted the matrix.

## 4 DISCUSSION

Eschar tissue‐derived fibroblasts are believed to originate from resident cells that have migrated from surrounding tissues to the wound bed upon injury.^13^ Following debridement, some of these cells may remain at the wound site or re‐establish in or in close vicinity to the wound bed. The myofibroblast‐like phenotype of these cells impairs wound healing by promoting contraction and subsequent scarring.^12^, ^13^ Given the critical role of these cells in wound healing, this study characterised the molecular profile of eschar‐derived fibroblasts and assessed their suitability for FSE production to mimic the wound environment in vitro.

In the first part of this study, the eschar fibroblasts were characterised using microarray analysis and compared to dermal fibroblasts. The pathway analysis revealed significant differences between the two cell types, particularly in the ECM receptor interaction, focal adhesion and TGF‐beta signalling pathways. These pathways influence each other, and a detailed investigation of the genes that are either upregulated or downregulated in these pathways may provide a more comprehensive understanding of the underlying changes. For instance, upregulated *COL10A1*, *COL11A1* and *INHBA* in eschar fibroblasts may suggest increased ECM synthesis, while downregulated *COMP* and *THBS4* may indicate potential impairments in ECM organisation.^30^, ^37^ The combined effect of these differentially expressed genes may contribute to fibrosis, characterised by excessive collagen production and improper assembly. In contrast, dermal fibroblasts demonstrated higher expression of *COMP* and *THBS4*, suggesting better collagen fibril assembly and ECM organisation.^38^, ^39^ Unexpectedly, genes that were differentially expressed in microarray analysis exhibited similar expressions in the 3D FSE models; both eschar and dermal fibroblasts contributed comparably to ECM synthesis and degradation, as was confirmed by RT‐qPCR and IHC staining. These observed differences in gene expression between microarray and FSEs may be because of distinct culture environments. In FSEs, fibroblasts were co‐cultured with keratinocytes and crosstalk between them is known to have an influence on gene expression.^40^ It may also be due to potential gene expression changes in eschar fibroblasts over the 23‐day FSE culture period. In addition, the number of donors used in the microarray analysis was higher than that used for the FSEs, and different donors were included in each study.

Myofibroblasts play a crucial role in wound closure due to their ability to generate strong contractile forces. Typically, they disappear once the wound has healed. However, if they persist, they can cause wound contraction and delay the healing process.^41^ The observed alterations in ECM‐receptor and focal adhesion pathways suggest that these cells respond to mechanical changes caused by injury. This mechanical stimulus may potentially influence the formation of the actin cytoskeleton and the stress fibres, ultimately contributing to the contractile phenotype.^42^ Considering their origin in the mechanically challenging wound environment, eschar fibroblasts were expected to have and retain a myofibroblast phenotype,^15^ including high *αSMA* expression, in both microarray analysis and FSEs. However, while the *actin alpha 1 (ACTA1)* expression was found to be similar between cell types in microarray analysis, eschar fibroblasts exhibited increased expression of *αSMA* at both the molecular and protein levels in FSEs. This may be because eschar fibroblasts used for the microarray were cultured in a 2D environment on cell culture flasks, while fibroblasts for FSEs were co‐cultured with keratinocytes in a 3D environment on a collagen matrix and exposed to growth factors.^43^ Alternatively, two‐day culture time for the microarray analysis may have been insufficient for *αSMA* expression.^44^ Moreover, it may also be important to mention the potential involvement of *INHBA*, a member of the TGF‐beta signalling pathway, which is highly expressed in eschar fibroblasts according to microarray data and is also take place in myofibroblast differentiation.^30^ While *INHBA* expression did not differ between cell types in FSE models after 23 days, it may have an early influence on eschar fibroblasts that lead to *αSMA* synthesis at a later stage.

Notably, αSMA protein staining was mainly observed underneath the epidermal–dermal interface. It has been shown that tension and mechanical forces generated by cells on the cell‐seeded surface stimulate αSMA expression.^45^ As can be seen from the H&E staining in Figure 3, the surface‐oriented layer has a higher cell density (including the epidermal layer) than the bottom layer. The mechanical forces generated by keratinocytes together with fibroblasts may stimulate eschar fibroblasts to differentiate into myofibroblasts. *αSMA* was expressed more intensely beneath the epidermis and eschar fibroblasts are more prone to synthesising αSMA. The density of cells and the resulting micromechanical forces and their impact on contraction were not analysed in this study but would be a significant point of interest to investigate further. It is also noteworthy that eschar fibroblasts reduced the dermal thickness more than dermal fibroblasts in Model 2 (Figure 3B), which may be attributed to the contractile forces generated by eschar fibroblasts.^46^

Following injury, complex repair processes initiate, including damaged tissue breakdown, cell migration and ECM reconstruction.^47^ Our microarray analysis indicated differences in ECM synthesis and remodelling in eschar fibroblasts. In this work, some genes that varied in microarray analysis were studied in FSEs as well. However, other genes not further evaluated in this study, such as *A2M*, an inhibitor of proteases involved in tissue degradation,^48^, ^49^, ^50^ was upregulated in eschar fibroblasts compared to dermal fibroblasts, whereas genes involved in cell–cell and cell–matrix interactions, cell migration and non‐apoptotic cell death, like *ROBO2* and *NPTX2*,^51^, ^52^, ^53^ were downregulated. Given the observed differences between microarray analysis and FSE models, these gene expressions may not differ between dermal and eschar fibroblasts in 3D models; nonetheless, further validation of significantly altered genes in 3D models may provide a deeper look into the wound environment.

Dermal fibroblasts are classified into two subtypes: papillary and reticular, each influencing wound healing in distinct ways. Papillary fibroblasts proliferate faster, and contribute to keratinocyte differentiation. In contrast, reticular fibroblasts are responsible for maintaining structural integrity, produce higher levels of type I collagen and exhibit greater contractility.^54^ In this study, dermal fibroblasts were isolated from split‐thickness autografts, which contain a mixture of both fibroblast subtypes, but most likely more papillary fibroblasts. It can be expected that eschar fibroblasts isolated from debrided tissue are primarily composed of reticular fibroblasts, as severe burn wounds damage the deeper layers of the skin where these cells are predominantly present and activated. Our findings revealed that eschar fibroblasts exhibited contractile behaviour compared to dermal fibroblasts, but showed similar behaviour for epidermal layer formation and collagen synthesis. This result could be different if a cell population of reticular fibroblasts, known for their higher contractile capacity, had been used instead of a cell population composed predominantly of papillary fibroblasts, which exhibit lower contractility.

Notably, αSMA expression appeared to be influenced by donor age. Eschar fibroblasts from younger donors exhibited higher levels of αSMA protein in FSEs. However, this observation needs to be strengthened by using a larger number of eschar donors.

In summary, we found many similarities between dermal and eschar fibroblasts, and both cell types are capable of forming a proper epidermis and dermis in full skin equivalents. Although it is known that donor age, post‐burn day and wound depth influence gene expression and induce variability,^55^ we clearly showed in microarray analysis that eschar fibroblasts lead to deviations from dermal fibroblast functions, for example ECM synthesis and organisation. Additionally, our findings from FSEs showed that eschar fibroblasts expressed *αSMA* and contracted the matrix, unlike dermal fibroblasts.

## 5 CONCLUSION

Eschar fibroblasts, with their distinct roles in ECM remodelling and contraction, differ from dermal fibroblasts originating from non‐wounded skin. This unique behaviour may be exploited for studying wound healing strategies by mimicking burn wound environment more closely in in vitro FSE model systems. This provides insights into the complex processes involved in wound repair and regeneration, thus paving the way for new treatment strategies.

## CONFLICT OF INTEREST STATEMENT

Gizem Coşar Kutluoğlu, Dominique Manikowski and Claudia Doberenz are current employees of MedSkin Solutions Dr. Suwelack AG, which manufactures and markets MatriDerm®. However, the opinions expressed in this research article were not influenced by the company. The other authors have no conflicts of interest to declare.

## Supporting information


**Table S1:** Genes used for the confirmation of microarray analysis. Gene symbol, gene name, accession number, forward and reverse primer sequences.
**Table S2.** Genes used for the RT‐qPCR of the FSEs. Gene symbol, gene name, accession number, forward and reverse primer sequences.
**Figure S1.** Epidermal and dermal layers were seen in all FSEs after 23 days of culture. Results show staining for cytokeratin 10, pan‐cytokeratin, type IV collagen, type III collagen and dermatan sulphate. In Model 2, type IV collagen and type III collagen staining, there is a small layer of dermis beneath the epidermis for Eschar‐FSE. The same orientation was used for the images with the epidermis upwards. *n* = 4 for each fibroblast type. Scale bar: 200 μm.
**Figure S2.** Staining of empty MatriDerm® as a control to demonstrate that it doesn't contain target antigens. Scale bar: 200 μm.
