# Supplementary material for: Comparison of dermal and eschar fibroblasts in full skin equivalents
Source: Wound Repair Regen. 2025 Feb 12;33(1):e70001. doi: 10.1111/wrr.70001 (PMC11822215; doi:10.1111/wrr.70001)
Supplement: Supplementary file 1 — Table S1: Genes used for the confirmation of microarray analysis. Gene symbol, gene name, accession number, forward and reverse primer sequences. Table S2. Genes used for the RT‐qPCR of the FSEs. Gene symbol, gene name, accession number, forward and reverse primer sequences. Figure S1. Epidermal and dermal layers were seen in all FSEs after 23 days of culture. Results show staining for cytokeratin 10, pan‐cytokeratin, type IV collagen, type III collagen and dermatan sulphate. In Model 2, type IV collagen and type III collagen staining, there is a small layer of dermis beneath the epidermis for Eschar‐FSE. The same orientation was used for the images with the epidermis upwards. n = 4 for each fibroblast type. Scale bar: 200 μm. Figure S2. Staining of empty MatriDerm® as a control to demonstrate that it doesn't contain target antigens. Scale bar: 200 μm. [file WRR-33-0-s001.docx]

Table S1: Genes used for the confirmation of microarray analysis. Gene symbol, gene name, accession number, forward and reverse primer sequences

| Gene Symbol | Gene Name | Accession Nr | Forward Primer | Reverse Primer |
| --- | --- | --- | --- | --- |
| GAPDH* | glyceraldehyde-3-phosphate dehydrogenase | NM_001256799 | GAAGTATGACAACAGCCTCAAG | GTGGCAGTGATGGCATGG |
| B2M* | beta-2-microglobulin | NM_213978 | GGCATTCCTGAAGCTGAC | ATGTCGGATGGATGAAACC |
| YWHAZ* | tyrosine 3 monooxygenase/tryptophan 5-monooxygenase activation protein zeta | NM_145690 | AGCAGAGAGCAAAGTCTTC | GCTTCTTGGTATGCTTGTTG |
| COL11A1 | collagen type XI alpha 1 chain | NM_080629 | CAGCAGCCTGGTATGATGTG | TTTCTGGACGCACAACCATC |
| INHBA | inhibin subunit beta A | NM_002192 | GAGTCAGGAACAGCCAGGAAG | AGAGGCGGATGGTGACTTTG |
| INHBB | inhibin subunit beta B | NM_002193 | TCTGCCTCCTCCTTCCACAC | GAATGCAGCAGGAGTTCACC |
| THBS4 | thrombospondin 4 | NM_003248 | CCCCAGGTCTTTGACCTTCTC | CTGCAGCTTGAAGGTGGAAATC |
| SHC3 | SHC adaptor protein 3 | NM_016848 | CAGTCTGAACCTGCGAACTC | TAGTCAGTTGTGTCCGGGTC |
| COMP | cartilage oligomeric matrix protein | NM_000095 | CAGATGCTTCGGGAACTGC | GTGATCTCCCTGACCTGCTG |

*Genes used as reference gene

Table S2: Genes used for the RT-qPCR of the FSEs. Gene symbol, gene name, accession number, forward and reverse primer sequences

| Gene Symbol | Gene Name | Accession Nr | Forward Primer | Reverse Primer |
| --- | --- | --- | --- | --- |
| GAPDH* | glyceraldehyde-3-phosphate dehydrogenase | NM_001256799 | GGCTGTGGGCAAGGTCATC | AGGAGTGGGTGTCGCTGTT |
| B2M* | beta-2-microglobulin | NM_004048.4 | CGCTACTCTCTCTTTCTGGC | GTCAACTTCAATGTCGGATGG |
| αSMA | actin alpha 2, smooth muscle | NM_001613 | TGATGGTGGGAATGGGACAA | CGTGAGCAGGGTGGGATG |
| COL1A1 | collagen type I alpha 1 chain | NM_000088.4 | CGGCTCCTGCTCCTCTTA | GTTTCTTGGTCGGTGGGTG |
| COL3A1 | [collagen type III alpha 1 chain](https://www.ncbi.nlm.nih.gov/gene/1281) | NM_000090.4 | AACTGCTCCTACTCGCCC | GTCCTCCTACTGCTACTCCA |
| hsp47 | heat shock protein 47 | NM_001235.5 | CAAGGGTGTGGTGGAGGTGA | AAAGGCGGTGGCGTGGAA |
| ELN | elastin | NM_001081752.3 | CAGGTGCGGTGGTTCCTCA | GACTCCAGGTTGCGGTCCC |
| TGFβ1 | transforming growth factor beta 1 | NM_000660.7 | GAAATCTATGACAAGTTCAAGCAGAG | GCTGAGGTATCGCCAGGAA |
| HAS1 | [hyaluronan synthase 1](https://www.ncbi.nlm.nih.gov/gene/3036) | NM_001297436.2 | GCCTGCGATACTGGGTAGC | ATGCGGTTGGTGAGGTGC |
| HYAL1 | [hyaluronidase 1](https://www.ncbi.nlm.nih.gov/gene/3373) | NM_033159.4 | AGGGCACAGGGAAGTCACA | CCAGAGCACCACTCCAGC |
| MMP2 | [matrix metallopeptidase 2](https://www.ncbi.nlm.nih.gov/gene/4313) | NM_001127891.3 | TGACGGAAAGATGTGGTGTG | TTGGTGTAGGTGTAAATGGGTG |
| MMP14 | [matrix metallopeptidase 14](https://www.ncbi.nlm.nih.gov/gene/4323) | NM_004995.4 | CTCAACCCAGGACTACCTCCC | CCTCACCCGCCAGAACCA |
| KRT10 | keratin 10 | NM_000421.5 | GGCGAGTCTTCATCTAAGGG | GCATCTGTAAATAATGGTCTGTG |
| IVL | involucrin | [NM_005547.4](https://www.ncbi.nlm.nih.gov/entrez/viewer.fcgi?db=nucleotide&id=1914825640) | TGTTCCTCCTCCAGTCAATACCC | CTCCTGTGGCTCCTTCTGCT |
| COL4A1 | [collagen type IV alpha 1 chain](https://www.ncbi.nlm.nih.gov/gene/1282) | NM_001303110.2 | GCACCGCTTCATCTCCTG | CTTCTTTCTCACCTCTTTCCTCTT |

*Genes used as reference gene


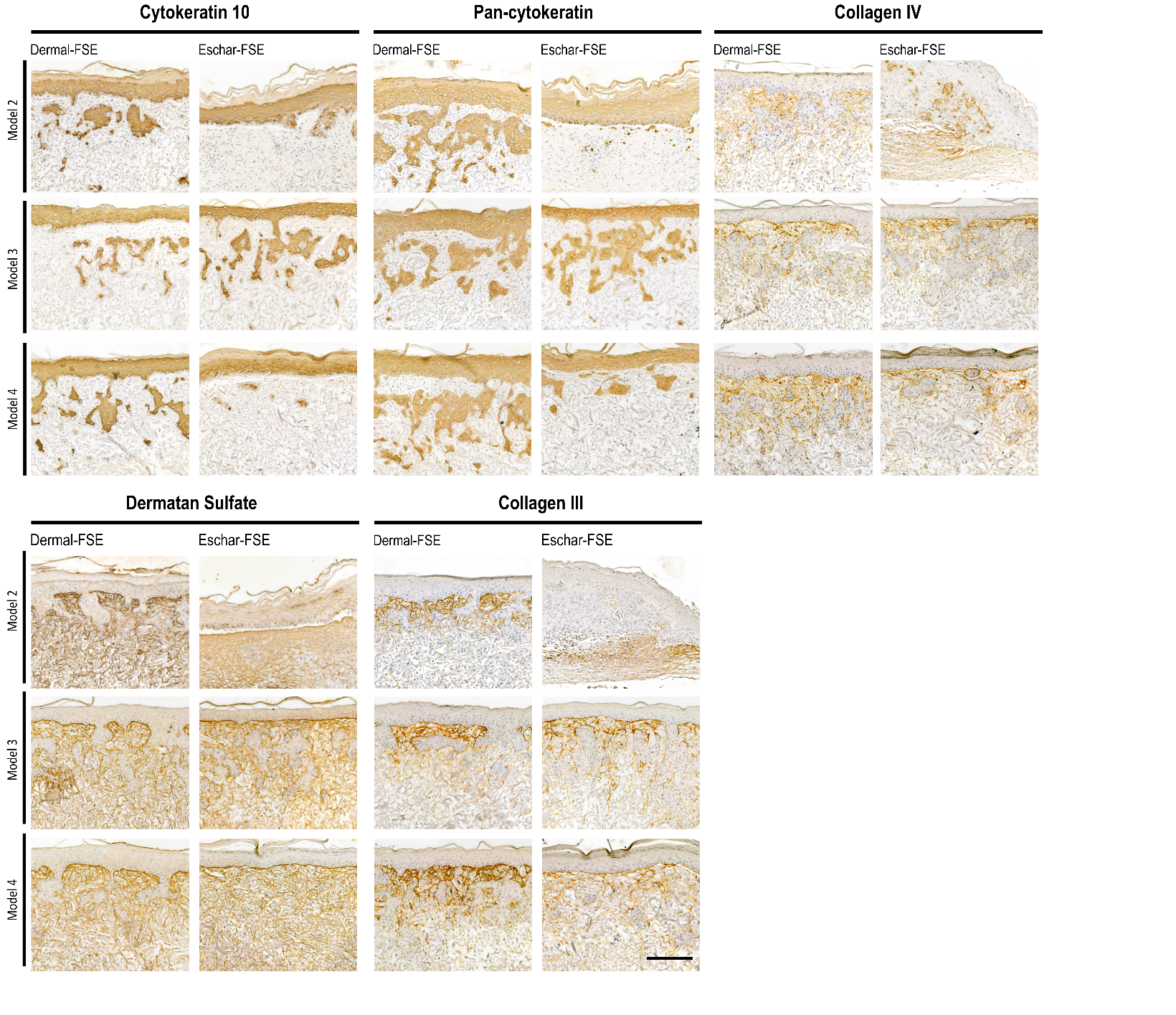


Fig. S1: Epidermal and dermal layers were seen in all FSEs after 23 days of culture. Results show staining for cytokeratin 10, pan-cytokeratin, type IV collagen, type III collagen and dermatan sulfate. In Model 2, type IV collagen and type III collagen staining, there is a small layer of dermis beneath the epidermis for Eschar-FSE. The same orientation was used for the images with the epidermis upwards. n=4 for each fibroblast type. Scale bar: 200 µm.


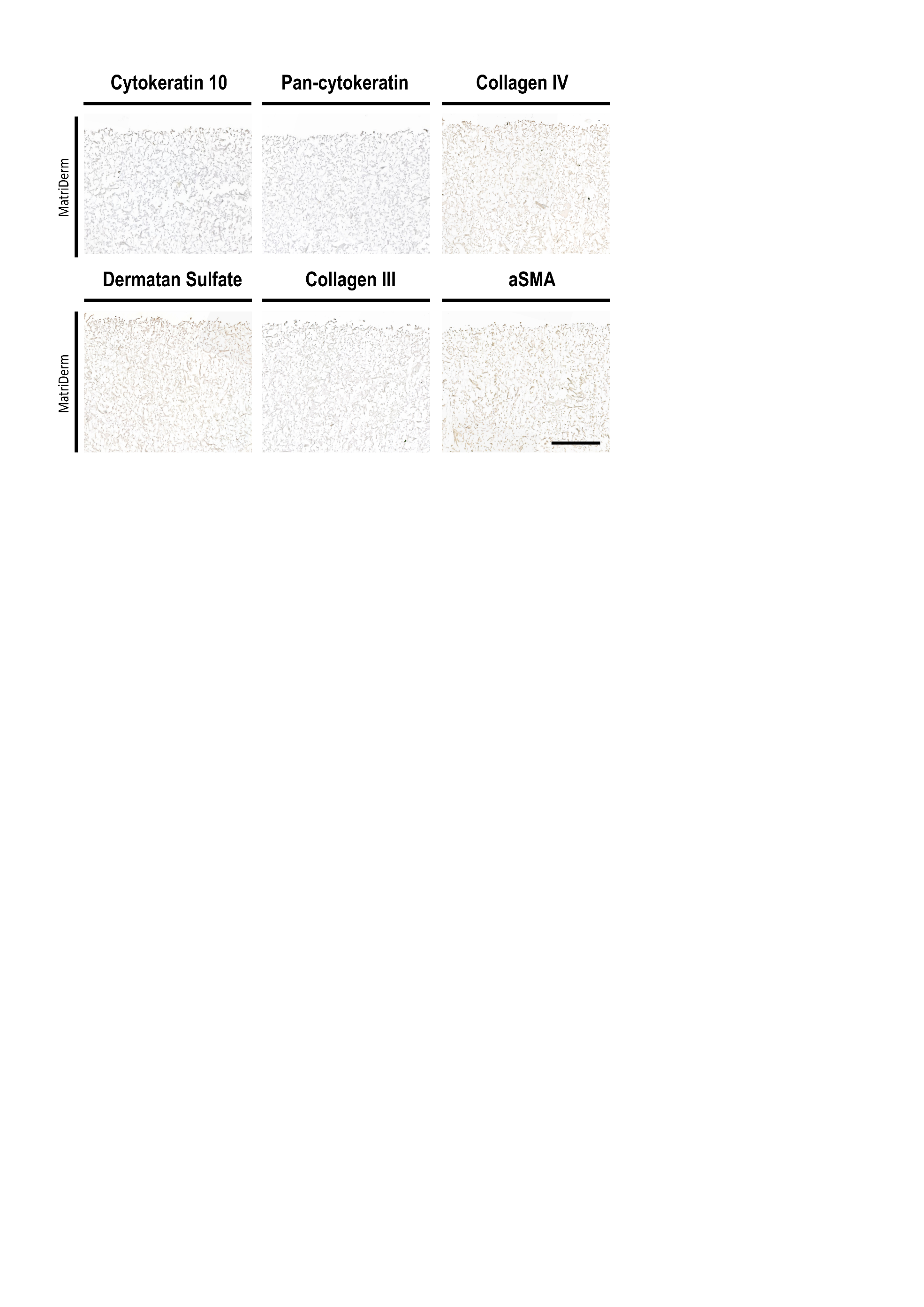


Fig. S2: Staining of empty MatriDerm as a control to demonstrate that it doesn’t contain target antigens. Scale bar: 200 µm.
